# Hepatorenal Syndrome: Aetiology, Diagnosis, and Treatment

**DOI:** 10.1155/2015/207012

**Published:** 2015-01-12

**Authors:** G. Low, G. J. M. Alexander, D. J. Lomas

**Affiliations:** ^1^Department of Radiology, Cambridge University Hospitals NHS Foundation Trust, Cambridge CB2 0QQ, UK; ^2^University of Alberta, Edmonton, AB, Canada T6G 2B7; ^3^University of Cambridge School of Clinical Medicine, Cambridge Biomedical Campus, P.O. Box 218, Cambridge CB2 0QQ, UK; ^4^Division of Gastroenterology & Hepatology, Cambridge University Hospitals NHS Foundation Trust, Cambridge CB2 0QQ, UK

## Abstract

Acute renal impairment is common in patients with chronic liver disease, occurring in approximately 19% of hospitalised patients with cirrhosis. A variety of types of renal impairment are recognised. The most important of these is the hepatorenal syndrome, a functional renal impairment due to circulatory and neurohormonal abnormalities that underpin cirrhosis. It is one of the most severe complications of cirrhosis with survival often measured in weeks to months. A variety of treatment options exist with early diagnosis and appropriate treatment providing the best hope for cure. This paper provides a comprehensive and up-to-date review of hepatorenal syndrome and lays out the topic according to the following sections: pathophysiology, historical developments, diagnostic criteria and limitations, epidemiology, precipitating factors, predictors, clinical and laboratory findings, prognosis, treatment options, prophylaxis, and conclusion.

## 1. Introduction

*Hepatorenal syndrome (HRS)* is a unique form of functional renal failure due to diminished renal blood flow, which occurs typically in kidneys that are histologically normal. It is a severe complication of advanced liver disease and characteristically affects patients with cirrhosis and ascites. Prognosis is poor with survival commonly measured in weeks to months. Due to the absence of recognised biomarkers, the diagnosis of HRS relies on a combination of clinical and laboratory criteria. Several treatment options exist, and early diagnosis and treatment provide the best hope of survival. In this paper, a comprehensive review of HRS is presented based on current knowledge. The information is organised according to the following sections: pathophysiology, historical developments, diagnostic criteria and limitations, epidemiology, precipitating factors, predictors, clinical and laboratory findings, prognosis, treatment options, prophylaxis, and conclusion.

## 2. Pathophysiology

**Figure 1:**
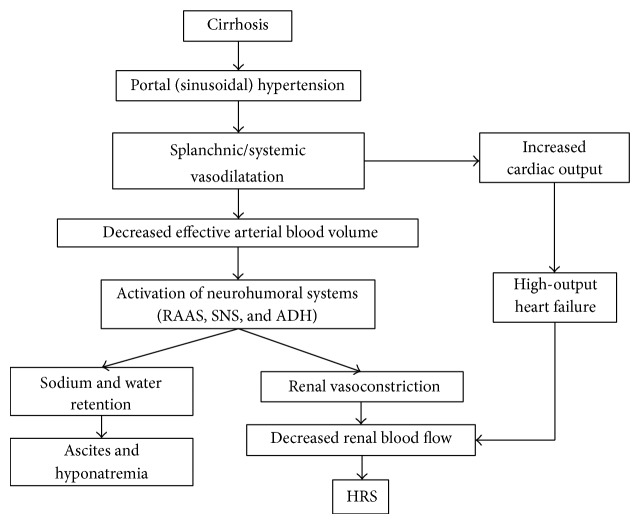
A flow diagram outlines the main pathophysiologic mechanisms involved in the development of HRS (reproduced with permission from “John Wiley and Sons,” Garcia-Tsao et al. [1]). RAAS—renin angiotensin aldosterone system; SNS—sympathetic nervous system; ADH—antidiuretic hormone; HRS—hepatorenal syndrome.

The peripheral arterial vasodilation theory is the most widely accepted explanation for the pathophysiology of HRS (Figure 1) [1], which proposes that splanchnic vasodilation that occurs as a consequence of portal hypertension with cirrhosis is the inciting factor for the development of HRS. Splanchnic vasodilation is mediated principally by nitric oxide but also to a lesser extent by other vasodilator substances such as carbon monoxide, glucagon, vasodilator peptides, and others. Splanchnic vasodilation sequesters blood in the splanchnic vascular bed leading to a reduced effective arterial blood volume (“*arterial underfilling*”). In compensated cirrhosis, cardiac contractility and cardiac output increase to counterbalance the reduction in systemic vascular resistance. This temporarily maintains the effective arterial blood volume. However, as the splanchnic vasodilation progresses the effective arterial blood volume ultimately declines. At a late stage, the cardiac output may also fall due to the development of cirrhotic cardiomyopathy. As the effective arterial blood volume declines, compensatory neurohormonal vasoconstrictor systems such as the renin-angiotensin-aldosterone system (RAAS), the sympathetic nervous system (SNS), and arginine vasopressin are stimulated. This causes sodium and water retention, leading in turn to ascites and hyponatraemia, as well as vasoconstriction of the renal, cerebral, and peripheral vascular beds. In the kidneys, local renal vasodilators such as prostaglandins are initially able to counterbalance the effects of the neurohormonal vasoconstrictor systems. Ultimately, this proves inadequate, as renal vasoconstrictor tone predominates. The end result of this process is a severe decline in renal blood flow leading to reduced glomerular filtration rate (GFR) and the development of HRS.

## 3. Historical Developments

The concept of HRS has its origins in important incremental discoveries that extend as far back as the 19th century. Frerichs (1861) and Flint (1863) reported an association between advanced liver disease and a type of renal impairment that is characterised by oliguria, the absence of proteinuria, and normal renal histology [2, 3]. By relating the renal impairment to disturbances in the systemic circulation, the authors offered the first pathophysiologic interpretation of HRS. In 1932, Helvig and Schutz introduced the term “*a liver and kidney syndrome,*” to describe a type of acute renal impairment that occurred following biliary surgery [4]. The definition of HRS evolved with time, and the term became synonymous with severe organ dysfunction that involved both the liver and kidneys simultaneously. In 1956, Hecker and Sherlock studied nine patients with advanced liver disease and renal impairment characterised by oliguria, hyponatraemia, low urinary sodium excretion, and absence of proteinuria [5, 6]. All patients died in hospital and postmortem findings showed normal renal histology. By correlating these observations with other relevant findings (i.e., low blood pressure, high cardiac output, and highly oxygenised peripheral venous blood), the authors postulated that peripheral arterial vasodilation was the key underlying mechanism for the development of HRS [5, 6]. This theory was strengthened by the observation that noradrenaline and volume expansion performed in three patients led to a transient improvement in the renal function. In 1970, Epstein et al. demonstrated the importance of splanchnic and systemic vasodilation together with renal vasoconstriction as a foundational concept in the pathophysiology of HRS [7]. In 1972, Vesin described HRS as “*functional renal insufficiency in cirrhosis*” and noted that the disease was often terminal [8]. Koppel et al. in 1969 and Iwatsuki et al. in 1973 provided compelling evidence for the functional nature of HRS [9, 10]. Koppel et al. reported reversal of the renal dysfunction when kidneys belonging to six patients with advanced liver disease and HRS were transplanted into recipients with end-stage kidney disease and normal liver function [9]. Iwatsuki et al. reported that liver transplantation in three cirrhotic patients with HRS led to improved liver and renal function within two weeks of operation [10]. Investigations by Schroeder et al., Arroyo et al., and Ring-Larsen et al. contributed to the understanding of the role of neurohormonal vasoconstrictor pathways (RAAS and SNS) in the genesis of HRS [11–13]. Building on advances in the field, Schrier et al. proposed an updated hypothesis for the pathophysiology of HRS, the peripheral arterial vasodilation theory [14]. In 1978, a consensus conference met in Sassari, Italy, to define diagnostic criteria for HRS [15]. The Sassari criteria did not receive widespread acceptance and were considered restrictive by many practicing physicians [15]. Finally, 1996 marked a watershed, when the International Ascites Club (IAC) produced diagnostic criteria for HRS that were adopted internationally [15]. These were later revised in 2007 [16].

## 4. Diagnostic Criteria and Their Limitations

Due to the lack of specific biochemical or radiologic markers, the diagnosis of HRS is based on criteria for excluding other causes of renal impairment that may be found in cirrhosis. The criteria defined by the IAC are listed as follows.

*Diagnostic Criteria for HRS (Reproduced with Permission from “John Wiley and Sons,” Arroyo et al. [15])*

*Major Criteria*

- Chronic or acute liver disease with advanced hepatic failure and portal hypertension.
- Low GFR as indicated by serum creatinine > 1.5 mg/dL or 24 hr creatinine clearance < 40 mL/min.
- Absence of shock, on-going bacterial infection, and current or recent treatment with nephrotoxic drugs and absence of gastrointestinal fluid losses (repeated vomiting or intense diarrhoea) or renal fluid losses (weight loss > 500 g/day for several days in patients with ascites without peripheral oedema or 1000 g/day in patients with peripheral oedema).
- No sustained improvement in renal function (decrease in serum creatinine ≤ 1.5 mg/dL or increase in creatinine clearance to ≥ 40 mL/min) following diuretic withdrawal and expansion of plasma volume with 1.5 L of isotonic saline.
- Proteinuria < 500 mg/dL and no sonographic evidence of obstructive uropathy or parenchymal renal disease.

*Additional Criteria*

- Urine volume < 500 mL/day.
- Urinary sodium < 10 mEq/L.
- Urinary osmolality greater than plasma osmolality.
- Urine red blood cells < 50 per high power field.
- Serum sodium < 130 mEq/L.

*Revised Diagnostic Criteria for HRS (Reproduced with Permission from “BMJ Publishing Group Limited,” Salerno et al. [16])*

- Cirrhosis with ascites.
- Serum creatinine > 133 *μ*mol/L (1.5 mg/dL).
- No improvement in serum creatinine (decrease to a level of ≤ 133 *μ*mol/L) after ≥ 2 days with diuretic withdrawal and volume expansion with albumin; the recommended dose of albumin is 1 g/kg of body weight/day up to a maximum of 100 g/day.
- Absence of shock.
- No current or recent treatment with nephrotoxic drugs.
- Absence of parenchymal kidney disease as indicated by proteinuria > 500 mg/day, microscopic haematuria (>50 red blood cells per high power field), and/or abnormal renal ultrasonography.

The revised criteria incorporate several new iterations which include (i) removal of creatinine clearance, (ii) recognition that on-going bacterial infection, in the absence of septic shock, no longer excludes a diagnosis of HRS, (iii) preference for the choice of albumin rather than saline for plasma expansion, and (iv) removal of the minor diagnostic criteria [16].

The IAC classifies HRS into type 1 and type 2. Type 1 HRS is a rapid progressive renal impairment defined by doubling of the serum creatinine to a level > 2.5 mg/dL or > 226 *μ*mol/L in less than two weeks [16]. Type 2 HRS is moderate renal impairment (serum creatinine > 1.5 and up to 2.5 mg/dL or > 133 and up to 226 *μ*mol/L) with a steady progressive course that evolves over weeks to months [16]. Acute deterioration in circulatory, renal, and hepatic function is characteristic of type 1 HRS, while these abnormalities develop more gradually in type 2 HRS. Type 1 HRS is often associated with a precipitating factor, while type 2 HRS typically develops de novo in patients with refractory ascites. Rarely, type 2 HRS may progress into type 1 HRS as a result of a triggering event. Prognosis is poor ranging from months in type 2 HRS to weeks in type 1 HRS.

The IAC diagnostic criteria have several shortcomings. The serum creatinine should be interpreted with caution in patients with cirrhosis [17–20]. These patients have lower baseline serum creatinine than normal due to (i) reduced endogenous creatinine production related to decreased hepatic synthesis and decreased muscle mass from malnutrition, (ii) medication related increased tubular secretion of creatinine, (iii) fluctuations in serum creatinine in patients with cirrhosis and large volume ascites (e.g., following diuretic therapy or paracentesis with volume expansion), and (iv) laboratory based underestimations of serum creatinine due to interactions with bilirubin [17–20]. As such, creatinine based measurements run the risk of overestimating renal function and underestimating the severity of renal impairment, which raises two important considerations. Firstly, a need exists to develop more accurate laboratory and imaging biomarkers of renal function. Cystatin C and NGAL (neutrophil gelatinase-associated lipocalin) have been advocated as renal biomarkers but are expensive and not widely available. As yet, standard imaging tests have proven unreliable for detecting HRS and differentiating it from other types of renal impairment. Secondly, expert consultation and consensus are required to determine if the serum creatinine threshold (>1.5 mg/dL) for HRS should be lowered to allow patients to be diagnosed and treated while being at an earlier stage [21].

Current IAC criteria do not consider the clinical scenario of HRS developing in patients with underlying chronic renal disease. There is an increasing realisation that patients that fit this category do exist but go unrecognized according to the definitions of the existing criteria [21, 22]. Munoz proposes that patients with HRS superimposed on chronic renal disease be categorised as having type 3 HRS [22]. Finally, it should be acknowledged that adherence to the IAC criteria is not always possible. As HRS is a diagnosis of exclusion, the diagnostic pathway can be complex at times, labour intensive, and prone to error. Some patients that do not fulfil the full IAC diagnostic criteria may be treated as having “*presumed*” HRS, based on the index of clinical suspicion. Three studies highlight these challenges. Salerno et al., in a study involving 253 patients with cirrhosis and renal failure, found that the diagnosis of HRS could only be “*presumed*” in 36% (*n* = 42) as not all the IAC criteria could be met [23]. In 17% (*n* = 20), diuretic therapy was tapered but not stopped and in 6% (*n* = 7) urinalysis could not be performed due to oligo-anuria or showed red blood cells and/or proteins due to bladder catheterisation or due to previous parenchymal renal disease, while in 7% (*n* = 8) ultrasound showed preexisting renal abnormalities [23]. Interestingly, the authors found no significant differences in the clinical characteristics, clinical-pathologic scores, or outcomes between presumed cases compared with cases that met the full criteria [23]. Servin-Abad et al. performed a retrospective analysis on 140 patients diagnosed with HRS from 1996 to 2004. The authors found that only 41 patients (29.3%) met the IAC criteria [24]. Causes of misdiagnosis included parenchymal renal disease (15.2%), acute tubular necrosis (ATN) (35.4%), active sepsis (34.3%), drug induced renal disease (4%), and others (11.1%) [24]. Watt et al. found that only 59% of patients with a clinical diagnosis of HRS met IAC criteria [25].

## 5. Epidemiology

*Incidence*. In a study in 1993, prior to the introduction of IAC criteria, Gines et al. reported that HRS had an incidence of 18% at one year and 39% at five years in patients with cirrhosis and ascites [26]. In a 2010 study utilising the revised diagnostic criteria, Montoliu et al. evaluated the incidence of functional renal impairment in 263 consecutive cirrhotic patients with ascites [27]. The authors found that 49% of patients developed functional renal impairment during follow-up (mean follow-up of 41 months). The annual incidence of HRS was 7.6% (*n* = 20) (type 1 = 7, type 2 = 13).

*Prevalence*. The prevalence of HRS (utilising the revised diagnostic criteria) in patients with cirrhosis and ascites ranged from 13 to 45.8%. Salerno et al. performed a prospective study of 253 consecutive patients with cirrhosis and renal impairment admitted to 21 Italian hospitals [23]. The prevalence of HRS was 45.8% (*n* = 116) (30% type 1 and 15.8% type 2). A prospective study by Martin-Llahi et al. of 562 consecutive patients with cirrhosis and renal impairment admitted to a single institution found HRS prevalence of 13% [28]. A prospective study by Thabut et al. of 100 consecutive patients with cirrhosis and renal impairment admitted to five French hospitals found HRS prevalence of 27% [29]. Retrospective studies suggest that HRS is present in 17% of cirrhotic patients admitted to hospital with ascites and in >50% of patients dying from end-stage liver failure [30, 31].

*Age*. Most patients with HRS are in their sixth or seventh decade. Salerno et al. reported the mean age as 62 ± 1.2 years (type 1 HRS) and 68 ± 1.6 years (type 2 HRS) [23], while Martin-Llahi et al. reported a mean age of 60 ± 12 years [28]. A pooled analysis by Garcia-Tsao et al. in 509 HRS patients from 14 studies found a mean age of 54 years [1].

*Gender*. There is a male preponderance, which is reflective of the gender balance of the underlying cirrhosis. Salerno et al. reported that 76.3% of type 1 HRS and 70% of type 2 HRS patients were males [23], similar to Martin-Llahi et al. (76.7%) [28] and Garcia-Tsao et al. [1].

*Aetiology of Cirrhosis*. Salerno et al. identified the aetiology of cirrhosis as* alcohol* (type 1 HRS 46.1%; type 2 HRS 55%),* viral* (type 1 HRS 31.6%; type 2 HRS 40%),* alcohol* +* viral* (type 1 HRS 10.5%; type 2 HRS 2.5%), and* others* (type 1 HRS 11.8%; type 2 HRS 2.5%) [23]. According to the pooled analysis by Garcia-Tsao et al., alcohol-related cirrhosis was the underlying aetiology in 57% (40 to 78%, interquartile range, IQR) [1].

## 6. Precipitating Factors

HRS develops on the background of advanced liver disease, as highlighted by the high mean Child-Pugh (CP) score of 11.2 (11-12; IQR) [1]. Cirrhosis is the most common underlying liver disease but other aetiologies include fulminant hepatic failure and severe acute alcohol-related hepatitis [32]. The frequency of HRS in fulminant hepatic failure and severe acute alcohol-related hepatitis has been reported to be as high as 55% and 30%, respectively [22, 33, 34]. HRS may occur spontaneously (typically in type 2 HRS) or may be triggered by a precipitating factor (in >70% of cases of type 1 HRS) [35].

The most common precipitating factor is spontaneous bacterial peritonitis (SBP). SBP refers to infection of ascitic fluid (typically by enteric Gram-negative bacteria) in the absence of a specific intra-abdominal source for the sepsis. SBP has a close chronologic and pathologic connection with HRS where it typically precedes its onset. Follo et al. [36] found that SBP precipitated HRS in 28% of cases despite appropriate treatment and resolution of infection. Renal impairment was transient in 21 cases (32%), stable in 26 cases (40%), and progressive in 18 cases (28%). SBP may trigger HRS via two postulated mechanisms [35, 37]: (i) release of proinflammatory cytokines (interleukin-6 and tumour necrosis factor) and endotoxins leading to increased production of nitric oxide and other vasodilator substances and (ii) sepsis-induced cardiomyopathy leading to decreased cardiac output.

The second most common precipitating factor for HRS is large volume paracentesis (LVP) without plasma expansion. LVP exacerbates the hyperdynamic circulation in cirrhosis, which leads to progressive systemic vasodilation and arterial underfilling. Gines et al. found that LVP (4–6 L/day), performed without intravenous albumin replacement, precipitated HRS in 21% of 53 cases [38]. In contrast, there were no cases of HRS when LVP was performed with intravenous albumin replacement. Cárdenas et al. found that renal impairment occurred in 11% of 175 of cirrhotic patients that experienced gastrointestinal bleeding [39]. In these patients, the aetiology was ATN not HRS. Gastrointestinal bleeding may induce a systemic inflammatory response associated with activation of proinflammatory cytokines that stimulate nitric oxide and other vasodilator substances. Furthermore, gastrointestinal bleeding increases susceptibility to infection, a vicious cycle that may generate further cytokines release, and rebleeding. Certain medications such as nonsteroidal anti-inflammatory drugs (NSAIDs) can precipitate HRS in those with borderline renal function. Renal vasoconstriction is initially counterbalanced by increased renal production of vasodilating prostaglandins (e.g., renal prostaglandin E2 and prostacyclin). NSAIDs can inhibit renal prostaglandin synthesis and thus aggravate renal vasoconstriction. Intravascular volume depletion by injudicious diuretic use has also been considered as a potential trigger for HRS; however, evidence to support this is lacking [32]. Biliary obstruction may precipitate HRS due to the action of bile acids and oxidative stress from free radical-induced tissue damage. Bile acids can alter the renal handling of electrolytes and water by blocking the sodium-hydrogen antiport protein [40]. Oxidative stress promotes the formation of a variety of vasoconstrictor substances including endothelin-1, cysteinyl leukotrienes, and F2-isoprostanes [40].

## 7. Predictors of HRS

Several studies have been performed to investigate potential predictors for HRS. Gines et al., in a study of 234 patients, identified 16 variables that may be useful as predictors of HRS on univariate analysis [26]. The variables included hepatomegaly, oesophageal varices, history of ascites, nutritional status, GFR, blood urea nitrogen, serum sodium and potassium, plasma renin activity, plasma noradrenaline, serum and urinary osmolality, urinary sodium excretion, free water clearance after a water load, and mean arterial pressure (MAP). However, only three independent variables, absence of hepatomegaly, high plasma renin activity, and low serum sodium, were found to be predictive of HRS on multivariate analysis. Montoliu et al., in a study of 263 patients, found that older age, high baseline serum creatinine, and a high CP score were independent predictors for HRS on multivariate analysis [27]. These variables may reflect the longer duration of the liver disease and the greater severity of the liver and renal impairment. Platt et al., in a study of 180 patients with nonazotemic liver disease, reported that the resistive index (RI) of the intrarenal arteries on Doppler ultrasound predicted the development of renal dysfunction, including HRS [41]. Renal dysfunction developed in 55% of patients with an elevated RI at baseline (≥0.7), including 6% of patients with a normal RI. HRS developed in 26% of patients with elevated baseline RI and 1% of patients with normal baseline RI. Patients that went on to develop HRS had higher baseline RIs (0.77) than patients that developed non-HRS renal impairment (0.72, *P* < 0.05) or patients with preserved normal renal function (0.65, *P* < 0.01). The RI may be regarded as a barometer of the intrarenal vascular tone and this is elevated in HRS due to increased vasoconstrictor activity.

## 8. Clinical and Laboratory Findings

HRS does not have specific clinical findings. Its physical manifestations broadly reflect the underlying advanced liver disease, renal impairment, and circulatory abnormalities present. Clinical findings of advanced liver disease include hepatomegaly, ascites, stigmata of portal hypertension (e.g., gastroesophageal varices, caput medusa, hepatic encephalopathy, etc.), jaundice, pruritus, coagulopathy, gynaecomastia, finger clubbing, palmar erythema, spider naevi, and constitutional disturbances such as weakness, fatigue, anorexia, and poor nutritional status. Patients with type 1 HRS are more severely affected than patients with type 2 HRS. Acute oliguria is typically present in type 1 HRS, while the urine output shows a more gradual decline in type 2 HRS. Circulatory disturbances include a hyperdynamic circulation and reduced systemic vascular resistance. This may manifest clinically as low MAP, low jugular venous pressure, tachycardia, a bounding pulse, and wide pulse pressure. The following laboratory findings are suggestive of HRS [26]: elevated plasma renin activity, elevated plasma noradrenaline activity, hyponatraemia, hyperkalaemia, elevated blood urea nitrogen, decreased plasma osmolality, elevated urinary osmolality, and decreased urinary sodium excretion. Serum abnormalities that reflect the severity of the liver disease include hyperbilirubinemia, hypoalbuminemia, and prolonged prothrombin time.

## 9. Prognosis

**Figure 2:**
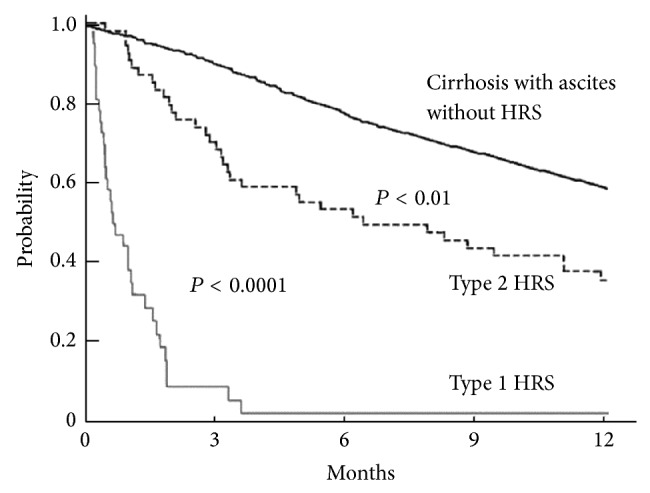
Probability of survival in cirrhotic patients with different types of renal impairment: nonazotemic patients (bold continuous line), type 2 HRS (dotted line), and type 1 HRS (continuous line) (reproduced with permission from “BMJ Publishing Group Limited,” Salerno et al. [16]).

HRS is one of the most lethal complications of cirrhosis (Figure 2). Prognosis is invariably poor ranging from months in type 2 HRS to weeks to months in type 1 HRS [42, 43]. Studies have been performed to define clinical parameters that track survival in HRS. In 2005, Alessandria et al. studied 105 patients with cirrhosis and HRS (39% type 1 and 61% type 2) [42] and found that HRS type and MELD (model for end-stage liver disease) score were variables that associated independently with survival on multivariate analysis. MELD is an internationally recognised scoring system developed for patients with advanced liver disease that is a predictor of three-month mortality and determines priority listing for liver transplantation [42, 44]. It is calculated according to the following formula: 9.6 × log⁡_e_ (creatinine mg/dL) + 3.8 × log⁡_e_ (bilirubin mg/dL) + 11.2 × log⁡_e_ (international normalised ratio, INR) + 6.4 [42]. Patients with type 1 HRS had a MELD score ≥ 20 and a median survival of one month [42]. For type 2 HRS, the median survival was 11 months for a MELD score < 20 and three months for a MELD score ≥ 20 [42]. Schepke et al. reported similar findings in a 2006 study that involved 88 patients with cirrhosis and renal failure [43], some with HRS (17–39.8% type 1, 22.7% type 2), and non-HRS renal impairment. On multivariate analysis, the authors found that type 1 HRS and the MELD score were independent variables associated with survival. The mean MELD score was higher in patients with HRS than in patients with non-HRS renal impairment (23.8 versus 18.3, *P* = 0.002) [43]. The estimated survival time was 3.4 months for type 1 HRS, 10.9 months for type 2 HRS, and 16.1 months for non-HRS renal impairment [43]. In 2010, Yang et al. performed a longitudinal assessment of prognostic factors in 103 patients with HRS (65% type 1 and 35% type 2) [45] and revealed that temporal changes (Δ) in four clinical parameters were associated with survival on time dependent multivariate analysis. These parameters included Δ MELD score simple, Δ serum creatinine, Δ serum bilirubin, and Δ serum albumin [45]. Δ MELD score simple was calculated according to the following formula: [3.8 × log bilirubin (mg/dL)] + 9.6 × log [creatinine (mg/dL) + 6.43] [45]. The authors found that Δ MELD score simple was superior to baseline and changes in MELD score in predicting prognosis. The authors also found that increasing serum creatinine and bilirubin affected survival adversely, while increasing serum albumin had a beneficial effect. In a 2012 study involving 68 patients with type 1 HRS, Martinez et al. reported that the aetiology of the liver disease, the serum creatinine at the time of initiation of treatment, and the urinary sodium were useful prognostic factors [46]. The aetiologies of the liver disease included (in decreasing order of survival) autoimmune hepatitis, cardiac, idiopathic, viral, viral + alcohol, alcohol, and neoplasia. The authors also found that higher serum creatinine on admission and a urinary sodium < 5 mEq/L were associated negatively with survival.

## 10. Treatment Options

*Vasoconstrictor Therapy*. This is the primary medical treatment for type 1 HRS. These drugs function by causing vasoconstriction of the grossly dilated splanchnic vascular bed resulting in increased systemic vascular return and increased MAP, which in turn suppresses the RAAS and SNS and improves renal perfusion. Albumin augments the potency of vasoconstrictor drugs by improving cardiac function and increasing the effective arterial blood volume. The Acute Dialysis Quality Initiative (ADQI) work group recommends the use of vasoconstrictor drugs combined with plasma expansion with albumin, as first line treatment for type 1 HRS [47]. Terlipressin is the vasoconstrictor drug of choice in Europe. It is a vasopressin analogue that acts on the V1 vasopressin receptors in vascular smooth muscle cells. The results of randomised controlled trials and meta-analysis suggest that the combination of terlipressin and albumin is effective in improving renal function in 40 to 50% of patients with type 1 HRS [48–50]. Patients with type 2 HRS may also benefit. Response to treatment is characterised by a decrease in the serum creatinine and an increase in the urine volume, serum sodium, and MAP. Median response time to treatment is 14 days [48]. Predictors of response include pretreatment bilirubin < 10 mg/dL and an increase of MAP ≥ 5 mmHg at day three of therapy [50–52]. Following withdrawal of treatment, HRS recurs in 20%, although retreatment is generally effective [16]. Terlipressin may cause organ and peripheral ischaemia and is contraindicated in patients with cardiovascular disease, cerebrovascular disease, and peripheral vascular disease. Significant complications are reported in 10 to 12% [16, 48–50]. It is unlikely that vasoconstrictor drugs improve survival beyond the short term. A multicentre randomised controlled trial comparing terlipressin and albumin to albumin alone in 46 patients with HRS showed improved renal function in the former group (43.5% versus 8.7%, *P* = 0.017), but no survival advantage in either group at three months (27% versus 19%, *P* = 0.7) [53]. A second multicentre randomised controlled trial in 56 patients with HRS comparing terlipressin to placebo and albumin found similar survival for both groups at 180 days (42.9% versus 37.5%, *P* = 0.8) [54]. Other vasoconstrictor drugs used in HRS include noradrenaline, midodrine, and octreotide. These have lower costs than terlipressin and, unlike terlipressin, are licensed for clinical use in North America. Noradrenaline and midodrine are *α*-adrenergic agonists, which act on the *α*_1_-adrenergic receptors in vascular smooth muscle cells. Octreotide is a long acting somatostatin analogue that inhibits glucagon and other vasodilator peptides. Only limited information is available about the efficacy of these alternative drugs.

*Transjugular Intrahepatic Portosystemic Shunt (TIPS)*. TIPS involves the insertion of an intrahepatic stent that connects the portal vein to the hepatic vein. This shunts portal blood into the systemic circulation, which reduces the portal pressure and increases the systemic venous return. In turn, this treats the arterial underfilling and the overactivity of the RAAS and SNS. Unfortunately, most patients with HRS are ineligible for TIPS due to contraindications (e.g., INR > 2, serum bilirubin > 5 mg/dL, CP score > 11, and cardiopulmonary disease). Furthermore, TIPS can aggravate the liver failure and precipitate encephalopathy.

Brensing et al. assessed the outcome following TIPS in 41 nontransplant HRS patients [55]. 31 patients received TIPS (14 patients, type 1, and 17 patients, type 2), and ten were excluded due to advanced liver disease. Renal function showed a gradual nonnormalized improvement within two weeks after TIPS. Overall survival following TIPS was 81% at three months, 71% at six months, 48% at 12 months, and 35% at 18 months. In contrast, seven of the ten non-TIPS patients died within three months. The authors concluded that TIPS could provide a survival benefit in well-selected HRS patients. Testino et al. performed TIPS in 18 type 2 HRS patients with refractory ascites that were awaiting liver transplantation [56]. The study found that TIPS improved the renal function and led to complete resolution of ascites in eight patients and partial resolution in ten patients. The authors suggested that TIPS may be used as a bridge to transplantation by preparing patients for surgery with improved renal function. Guevara et al. assessed the effects of TIPS on renal function and the vasoactive systems [57] and found improved renal function to nonnormalized levels in six of seven patients with type 1 HRS. The serum creatinine fell from 5 ± 0.8 to 1.8 ± 0.4 mg/dL by 30 days. Reduced activity of the RAAS and SNS was reflected by a reduction in serum renin, aldosterone, and norepinephrine levels. Testino et al. performed TIPS in nine patients with type 1 HRS and acute alcohol-related hepatitis [58], which resulted in a nonnormalized improvement in renal function (decreased serum creatinine and blood urea nitrogen and increased urine volumes). The serum creatinine fell from 5.2 ± 0.9 mg/dL to 1.6 ± 0.6 mg/dL by 30 days. Wong et al. evaluated the combination of medical therapy (midodrine, octreotide, and albumin) and TIPS in type 1 HRS [59]; serum creatinine decreased to < 135 *μ*mol/L for at least three days following medical therapy in ten patients. Half of the responders underwent TIPS and showed resolution of ascites with normalisation of renal function (GFR 96 ± 20 mL/min) and serum renin and aldosterone by 12 months.

The ADQI work group recommends that (i) TIPS should not be used as the first line treatment for type 1 HRS due to insufficient data and (ii) TIPS may be used in patients with type 2 HRS and refractory ascites [47].

*Extracorporeal Support Systems*.* Renal replacement therapy (RRT)* may be used to treat specific complications of renal impairment such as metabolic acidosis, hyperkalaemia, volume overload, and uraemic symptoms. It may have a role in patients who are unresponsive to vasoconstrictor drugs and where TIPS is contraindicated. In some circumstances, it may provide a bridge to liver transplantation. Side effects include hypotension, coagulopathy, and infection. Continuous venovenous haemofiltration is preferred to intermittent haemodialysis in unstable patients and those at risk of developing raised intracranial pressure. The ADQI work group recommends that RRT should be avoided in type 1 HRS patients unless there is an acute reversible component or an intention to pursue transplantation [47].

*Molecular adsorbent recirculating system (MARS)* is a modified dialysis technique for extracting albumin bound and water-soluble substances from the blood. This removes vasodilator substances such as nitric oxide, tumour necrosis factor, and cytokines, which are implicated in the pathogenesis of HRS. Mitzner et al. performed a randomised controlled study in type 1 HRS patients undergoing either MARS (eight patients) or standard treatment (five patients) [60] and found a significant reduction in the serum creatinine and bilirubin and a significant increase in the serum sodium and prothrombin activity. The MARS group showed improved short-term survival compared with the control group (survival was 37.5% at seven days and 25% at 30 days for MARS versus 0% at seven days for the control group). In a separate study involving eight subjects, Mitzner et al. found that MARS improved multiorgan function in type 1 HRS patients [61]. Wong et al. performed a study involving six type 1 HRS patients with refractory ascites that had failed vasoconstrictor treatment [62] and found that MARS was not associated with significant improvements in the GFR, neurohormonal levels, or systemic haemodynamics. In a study of 32 patients, Lavayssière et al. found that only 28% of type 1 HRS patients showed complete renal recovery after 28 days [63]. More recently, a large randomised controlled trial (RELIEF) involving 19 European centres comparing MARS with standard therapy in patients with acute-on-chronic liver failure [64] reported that while MARS provided temporary organ benefit (liver, kidney, and brain), it did not improve overall survival.

Prometheus is an extracorporeal technique involving fractional plasma separation and adsorption with haemodialysis for removing water-soluble and albumin bound substances. Rifai et al. found that Prometheus treatment in ten HRS patients led to improved serum creatinine, urea, bilirubin, bile acids, and ammonia concentrations [65]. Given the lack of a definitive survival benefit and high costs, the ADQI work group suggests that extracorporeal support systems should be limited to research protocols.

*Liver Transplantation*. Liver transplantation is the definitive treatment for HRS [66–72]. The five-year survival for HRS is 60% for patients that underwent liver transplantation compared with 0% for patients that did not undergo liver transplantation [66]. The use of the MELD scoring system, which allocates liver grafts according to the “sickest first” policy, has increased the proportion of HRS patients that have received a liver transplant. In addition to orthotopic liver transplantation (OLT) [40], some centres have also performed living donor liver transplantation (LDLT). Advantages of LDLT include increasing the donor pool, the possibility of planned surgery, shorter ischaemic duration, and younger donor age [73]. A potential pitfall is that the smaller graft may be insufficient to sustain hepatic function [68]. A report by Lee et al. involving 71 HRS patients (48 OLT and 23 LDLT) found comparable outcomes for both techniques [73]. The three-year survival was 85.3% for LDLT compared with 60.9% for OLT.

Patients with HRS have worse posttransplant outcomes than patients without HRS. This includes reduced short- and long-term survival, increased risk of bleeding and infection, and longer hospital stay [66, 67]. The five-year survival comparing patients with HRS that have received a liver transplant to patients without HRS that have received a liver transplant is 60% versus 68% (*P* < 0.03) [66, 67]. The pretransplant renal function is a major predictor of outcomes following transplantation, with less favourable outcomes for patients with pretransplant renal dysfunction [66, 72]. Restuccia et al. suggested that reversal of the renal dysfunction prior to transplantation improves posttransplant outcomes [68]. The study found that HRS patients treated with vasopressin analogues before transplantation had similar outcomes to controls with normal renal function [68]. Both groups had comparable survival (three-year survival 100% in HRS-treated group versus 83% in controls, *P* = 0.15), frequency of posttransplant renal dysfunction, and length of hospital stay.

Between 58 and 94% of patients with HRS demonstrate recovery of renal function after liver transplantation [74–76]. Factors associated with a failure of renal recovery include the time interval between onset of HRS and transplantation (≥ 4–6 weeks), dialysis for ≥ 8 weeks, and a serum creatinine of ≥ 2 mg/dL [74–77]. Such patients may benefit from simultaneous liver and kidney transplantation. The ADQI work group recommends that liver transplantation alone should be performed if the duration of type 1 HRS is < 4 weeks, while simultaneous liver and kidney transplantation should be performed if there is a risk that renal recovery will not occur [47].

## 11. Prophylaxis

Prophylactic treatment may be beneficial in reducing the risk of developing HRS. Sort et al. performed a randomised controlled trial in cirrhotic patients with SBP [78]. 126 patients were randomised to one of two treatment arms, albumin plus cefotaxime (antibiotic) or cefotaxime alone. The study found that patients that received albumin plus cefotaxime had a lower incidence of developing renal impairment than patients that received cefotaxime alone (10% versus 33%, *P* < 0.01). In-hospital and three-month mortality rates were significantly lower in the albumin plus cefotaxime group compared with the cefotaxime group (10% versus 29%, *P* = 0.01, and 22% versus 41%, *P* = 0.03). Pentoxifylline is a phosphodiesterase inhibitor with beneficial effects on renal function. Tyagi et al. performed a randomised controlled trial involving 70 patients with cirrhosis, ascites, and a baseline normal renal function [79]. Patients were randomised into two treatment arms, pentoxifylline or placebo. Baseline, one-month, three-month and sixth-month laboratory and clinical parameters were assessed. HRS developed in two patients in the pentoxifylline group (2.9%) compared with ten patients in the placebo group (14.3%). The six-month mortality was one in two HRS patients in the pentoxifylline group compared with three in ten HRS patients in the placebo group.

## 12. Conclusion

Over the last century, much has been learnt about the pathophysiology, clinical behaviour, and natural history of HRS. Standardised diagnostic criteria have been developed and implemented worldwide, allowing for more uniform diagnosis and consistent reporting of the disease. Limitations in the diagnostic criteria exist, but as yet, no reliable diagnostic marker exists for HRS. Future directions should include the development of an accurate diagnostic test for HRS. This is important as an earlier diagnosis and thus treatment is likely to improve survival. Several treatment options exist, but, at present, only liver transplantation offers a genuine hope for cure and longevity.
